# Enrichment of Cancer‐Associated Fibroblasts, Macrophages, and Up‐Regulated TNF‐α Signaling in the Tumor Microenvironment of CMS4 Colorectal Peritoneal Metastasis

**DOI:** 10.1002/cam4.70521

**Published:** 2024-12-30

**Authors:** Eirik Høye, Chakravarthi Kanduri, Annette Torgunrud, Susanne Lorenz, Bjørn Edwin, Stein G. Larsen, Åsmund A. Fretland, Vegar J. Dagenborg, Kjersti Flatmark, Christin Lund‐Andersen

**Affiliations:** ^1^ Department of Tumor Biology, The Norwegian Radium Hospital Oslo University Hospital Oslo Norway; ^2^ Institute of Clinical Medicine University of Oslo Oslo Norway; ^3^ Department of Informatics University of Oslo Oslo Norway; ^4^ Department of Core Facilities, The Norwegian Radium Hospital Oslo University Hospital Oslo Norway; ^5^ The Intervention Centre, Rikshospitalet Oslo University Hospital Oslo Norway; ^6^ Department of Hepato‐Pancreato‐Biliary Surgery, Rikshospitalet Oslo University Hospital Oslo Norway; ^7^ Department of Surgical Oncology, The Norwegian Radium Hospital Oslo University Hospital Oslo Norway

## Abstract

**Background:**

Metastatic colorectal cancer (mCRC) is the main cause of CRC mortality, with limited treatment options. Although immunotherapy has benefited some cancer patients, mCRC typically lacks the molecular features that respond to this treatment. However, recent studies indicate that the immune microenvironment of mCRC may be modified to enhance the effect of immune checkpoint inhibitors. This study aimed to explore the metastatic tumor microenvironment (TME) by comparing cell populations in colorectal liver (CLM), lung (mLu), and peritoneal (PM) metastases.

**Methods:**

RNA isolated from 20 CLM, 15 mLu, and 35 PM samples was subjected to mRNA sequencing and explored through TME deconvolution tools, consensus molecular subtyping (CMS), and differential gene expression and gene set enrichment analysis, with respect to the metastatic sites. Clinical data and *KRAS*/*BRAF* hotspot mutation status were also obtained for all the cases.

**Results:**

The cell type fractions in the TME were relatively similar between the metastatic sites, except for cancer‐associated fibroblasts (CAFs), B cells, endothelial cells, and CD4+ T cells. Notably, PM showed enrichment for CAFs and endothelial cells, consistent with distinct pathways associated with metastatic growth and progression in the peritoneal cavity. PM with the mesenchymal subtype, CMS4, had increased CAFs, endothelial cells, and macrophages, along with up‐regulated genes related to TNF‐α signaling via NF‐κB, EMT, and angiogenesis.

**Conclusions:**

Tumor samples from different metastatic sites exhibited a broadly similar TME in terms of immune cell composition, with some intriguing differences. Targeting CAF‐associated pathways, macrophages, and TNF‐α signaling through NR4A could represent potential novel therapeutic approaches in CMS4 PM.

## Graphical abstract

Immunotherapy is ineffective in mCRC; however, the efficacy could be significantly enhanced by modifying the tumor immune microenvironment. Comparison of immune cell populations and cell signaling in colorectal liver, lung, and peritoneal (PM) metastases using immune cell deconvolution tools and transcriptomic data, revealed enrichment of CAFs and CAF‐related secreting factors in the CMS4 PM microenvironment, that could facilitate intraperitoneal spread, immune suppression, and chemotherapy resistance. Targeting CAF‐associated pathways, M2 macrophages, and TNF‐⍺ signaling could be novel therapeutic strategies for CMS4 PM‐CRC, as they might alter the tumor immune interplay and improve immunotherapy response.

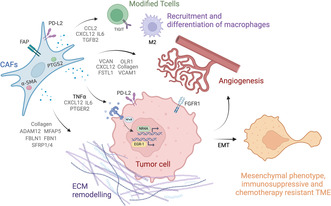

**Abbreviations**
CAFs: cancer‐associated fibroblasts
CD8^+^
: cytotoxic T cells
CLM: colorectal liver metastasis
CMS: consensus molecular subtypes
CRC: colorectal cancer
DEGs: differentially expressed genes
GSEA: gene set enrichment analysis
ICI: immune checkpoint inhibition
mCRC: metastatic colorectal cancer
mLu: colorectal lung metastasis
MSI: microsatellite instable
MSS: microsatellite stable
OS: overall survival
pCRC: primary colorectal cancer
PM: colorectal peritoneal metastasis
RIN: RNA integrity numbers
TPM: principle component analysis
TPM: transcripts per million
VST: varianceStabilizingTransformation

## 1 Introduction

Colorectal cancer (CRC) is one of the most frequent cancers worldwide, with over 1.93 million new cases and more than 940,000 deaths in 2020 [1]. While patients with localized primary cancers (pCRC) may be cured by surgical resection, only a limited proportion of patients with metastatic progression (mCRC) are eligible for curative surgical interventions. Palliative systemic chemotherapy is the most important treatment option in mCRC, which remains the main cause of CRC mortality [2]. Over the last decade, the introduction of immune checkpoint inhibition (ICI) has dramatically changed the outlook for large groups of patients with metastatic cancer [3]. However, mCRC rarely exhibits molecular features typically associated with response to ICI, with only 4%–5% being microsatellite instable (MSI), and responses to ICI have been poor in microsatellite stable (MSS) CRC [4].

Although MSS CRC is considered to be “immunologically cold,” the importance of the immune microenvironment in pCRC has been strongly supported by previous studies [5, 6, 7]. In seminal work, Galon and coworkers showed that a high density of cytotoxic T cells (CD8^+^) in the center of the tumor and in the tumor invasive margin was associated with a favorable overall survival (OS) in pCRC [5]. In the metastatic setting, however, much less is known, partly because of the limited availability of metastatic samples for analysis. We previously showed that in resectable MSS colorectal liver metastases (CLM), administration of cytotoxic chemotherapy was associated with a transient increase in tumor T cell infiltration [8]. The finding suggests that chemotherapy‐induced tumor cell death may trigger an immune response, in accordance with the theory of immunogenic cell death, and that the immune microenvironment of mCRC may be modified, possibly to improve responses to ICI [9]. In order to improve treatment responses and survival in mCRC, studies to improve our understanding of the metastatic tumor immune microenvironment are warranted.

With information from a cellular level based on methods such as flow cytometry, immunohistochemistry staining, or single‐cell sequencing often being unavailable, computational methods to estimate the immune‐cell composition from transcriptomic data generated from analysis of bulk tissue have been developed. In a recent benchmarking study, the capabilities and limitations of available methods were evaluated systematically by simulating bulk tissue from single‐cell RNA‐seq datasets of known cell types from the tumor microenvironment [10]. Deconvolution methods were benchmarked on predictive accuracy against ground‐truth datasets, demonstrating that computational deconvolution performed at high accuracy for well‐defined cell‐type signatures.

The study aimed to investigate the TME of mCRC, comparing samples from liver, lung, and peritoneal metastases obtained during curatively intended surgery. Next‐generation RNA sequencing data were explored using TME deconvolution, differential gene expression, and gene set enrichment analysis (GSEA), to assess differences in immune infiltration and cell signaling pathways between the metastatic sites. Associations with metastatic location and consensus molecular subtypes (CMS) were also performed.

## 2 Materials and Methods

### 2.1 Patient Samples

Metastatic tumor samples from surgical resection specimens were collected during curatively intended surgery for mCRC. Patients were included after written informed consent in three studies that were approved by the Regional Ethics Committee of South‐East Norway. CLM samples (*n* = 20) were collected as part of the OSLO‐COMET trial (NCT01516710). This was a randomized controlled trial comparing laparoscopic and open liver surgery for patients with resectable CLM, as described in [8]. Patients with peritoneal (PM, *n* = 35; NCT02073500) and lung metastases (mLu, *n* = 15; REC ID# S‐06402b) were included in observational studies [11]. PM samples were selected to equally represent key hotspot mutations in *KRAS* and *BRAF* oncogenes, or *KRAS*/*BRAF* wild‐type (Table 1). Tissue samples were collected at the time of surgery, immediately snap‐frozen in liquid nitrogen, and stored at −80°C.

**TABLE 1:** Clinical and molecular characteristics of patients.

Variable		CLM	mLu	PM***
Gender (*n*, (%))	Male	8 (40)	9 (60)	24 (69)
	Female	12 (60)	6 (40)	11 (31)
Age*; years (median, min‐max)		65 (48–80)	59 (34–76)	62 (31–76)
Overall survival**; months (median, min‐max)		61 (12–95)	85 (17–110)	28 (2–96)
Primary tumor location (*n*, (%))	Right colon	4 (20)	0 (0)	15 (43)
	Left colon	7 (35)	8 (53)	17 (49)
	Rectum	9 (45)	7 (47)	3 (8)
Consensus molecular subtype (CMS) (*n*, (%))	CMS1	0 (0)	0 (0)	8 (23)
	CMS2	19 (95)	15 (100)	12 (34)
	CMS3	0 (0)	0 (0)	2 (6)
	CMS4	1 (5)	0 (0)	11 (31)
	Not determined	0 (0)	0 (0)	2 (6)
Mutation status (*n*, (%))	*KRAS* mutated	8 (40)	7 (47)	12 (34)
	*BRAF* mutated	1 (5)	0 (0)	13 (37)
	WT	11 (55)	8 (53)	10 (29)

*Note:* Colorectal liver metastasis (CLM); lung metastasis (mLu); peritoneal metastasis (PM); *, at the time of metastasis surgery; WT, wild‐type *KRAS* and *BRAF*; **, from the time of metastasis surgery; ***, PM cases were selected to numerically equally represent mutated *KRAS*, *BRAF*, and WT cases. CMS status for two PM patients was not determined.

### 2.2 Tissue Processing, mRNA Sequencing, and Bioinformatics Pipeline

Frozen sections were generated from the collected metastatic tumor tissue and hematoxylin and eosin‐stained slides were evaluated by a pathologist. Samples with a minimum of 25% tumor tissue (median 50%; full range 25%–100%) were processed as described in [12]. Total DNA and RNA were extracted using the Allprep DNA/RNA/miRNA Universal Kit (Qiagen, Düsseldorf, Germany; Cat. No. 80224), using 20–30 mg tissue per sample as input. RNA concentration and purity were measured using the Nanodrop 2000 spectrophotometer (Thermo Fisher, Waltham, Massachusetts, USA). RNA integrity numbers (RIN) were estimated with Bioanalyzer RNA 6000 Nano kit (Agilent Technologies, Santa Clara, California, USA). Total RNA was diluted to 50 ng/μL in 20 μL, and mRNA sequencing libraries were prepared using the TruSeq Stranded mRNA kit (Illumina, San Diego, California, USA) following the vendor's protocol. The mRNA sequencing data was generated from two sequencing runs: GCF0506 and GCF0620. GCF0506 was sequenced on a NextSeq500 machine, while GCF0620 was sequenced on a NovaSeq6000 machine (both from Illumina, San Diego, California, USA). Transcription quantification was carried out using Salmon v = 1.4.0 [13] in selective alignment mode with a decoy‐aware transcriptome using the default k‐mer length of 31. The transcriptome of genome reference consortium human build 38 patch release 13 (GRCh38p13) including alternative loci was used for building the transcriptome index. For optimizing the abundance estimates, Salmon's variational Bayesian EM algorithm was used. In addition, Salmon's built‐in models to correct for the sequence‐specific biases and fragment‐level GC biases were used.

### 2.3 Analysis of Technical Variation Between Gene Expression From Two Sequencing Runs

Of the two sequencing runs, GCF0506 included only PM samples (*n* = 30), while GCF0620 included CLM (*n* = 20), mLu (*n* = 15), and PM (*n* = 5). Principal component analysis (PCA) plots were made using the FactoMineR PCA() function. To assess the presence of any technical variation in gene expression and cell composition derived from the samples being analyzed in different sequencing runs, the PM samples (30 + 5 samples analyzed in separate runs) were used. Count matrices of ~20,000 protein‐coding genes were normalized with the varianceStabilizingTransformation (VST) function from the DESeq2 v1.30.1 Bioconductor package [14]. Differential gene expression comparison between PM samples from batches GCF0620 and GCF0506 was conducted using DESeq2 v = 1.30.1 [14].

### 2.4 Comparison of Deconvolution Methods for Assessment of TME Cell Composition

Several tools, based on different underlying algorithms, are available for cell deconvolution, and a concordance analysis was performed to compare estimated cell fractions between three commonly used tools EPIC [15], quanTIseq [16], and CIBERSORT (absolute mode) [17] using the datasets in this study. Concordance between rank transformed cell fraction estimates of EPIC and quanTIseq showed moderate concordance between Β cell, CD4^+^ cell, and NK cell estimated fractions, but no concordance between CD8^+^ cell and macrophage estimated fractions (Figure S1A), while there was poor concordance between CIBERSORT and the two other methods. A previously published benchmarking study simulating analysis of bulk tissue recommended the EPIC tool for general deconvolution tasks based on the predictive correlation with single‐cell sequencing data [10]. For subsequent analyses, EPIC was chosen as the main tool. All cell deconvolution methods were run through the immunedeconv R package v = 2.0.3 [10], with EPIC v = 1.1.0, using RNA‐seq TPM files as input.

### 2.5 CMS Classification

CMS classification of samples from GCF0620 was performed using the CMSclassifier package [18] provided by [19]. CMS data for samples included in GCF0506 were available from [11].

### 2.6 Analysis of Hotspot Mutations in *KRAS* and *BRAF*

DNA‐based hotspot mutation data of PM samples and five CLM samples were available from [11] and [20], respectively. Tumor DNA was analyzed by targeted next‐generation sequencing with either Ion AmpliSeq Cancer Hotspot panel v2 (HS, *n* = 24) or Oncomine Comprehensive panel v3 (Onc, *n* = 16) (Thermo Fisher Scientific), both covering hotspot mutations in *KRAS* and *BRAF*. Variants were called, annotated, and filtered using Torrent Suite Variant Caller/ANNOVAR based in‐house pipeline (HS) and Ion Reporter Software V.5.10 (Onc) (Thermo Fisher Scientific) and manually reassessed using Integrative Genomics Viewer. Variant detection from RNA sequencing reads was conducted for mLu and CLM samples (GCF0620) in the absence of DNA mutation data. Human genome release 38 (GRCh38.p13) reference genome was used for generating genome index for STAR alignment tool [21] with corresponding annotation version from GENCODE (gencode 38). STAR was further used for mapping the reads from RNA‐seq libraries to the indexed genome to generate BAM files that are sorted by coordinate. The generated BAM files were uploaded to Integrative Genomics Viewer (IGV) for assessment of hotspot mutations in *KRAS* (codon12/13) and *BRAF* (V600E). Variants were detected with an allelic fraction ranging from 30% and 100% and an average coverage of 42×.

### 2.7 Gene Set Enrichment Analysis (GSEA)

Differentially expressed genes (DEGs) between PM, CLM, and mLu samples that were tissue‐specific for liver and lung (from gene expression signatures in The Human Protein Atlas) were excluded prior to analysis. The remaining DEGs were subjected to GSEA using the web‐based tool Enrichr [22] and the Hallmark gene set 2020 from the molecular signatures database (MSigDB).

### 2.8 Statistical Analyses

Fisher's exact test (R v = 4.2.2) was used to compare the distribution of gender, primary tumor location, and CMS status between CLM, mLu, and PM. Spearman correlation was used for concordance analysis of TME cell types using different deconvolution tools. Wilcox ranked sum test was used when comparing cell fractions. Benjamini‐Hochberg (BH) correction for multiple testing was used to identify cell types with robust variation between tissue type and CMS class, with an adjusted *p*‐value threshold of 0.05 considered statistically significant. DESeq2 v = 1.30.1 Bioconductor package [14] was used for differential expression analysis, with BH adjusted *p*‐value threshold < 0.1. Student's t‐test was used for comparison of gene expression between PM CMS4 and CMS1‐3. OS was calculated from date of resection to date of death, with censoring time set at 5 years. The Kaplan–Meier method was used to analyze OS, and the log‐rank test was used to compare survival between metastatic sites with *p*‐values < 0.05 being considered significant.

## 3 Results

### 3.1 Clinical and Molecular Parameters

Key clinical and molecular parameters are summarized in Table 1. The age and gender distributions were relatively similar between the three metastatic locations. The majority of the primary tumors were located in the left colon and rectum for the CLM and mLu groups, while for PM, a large proportion of the primaries were in the right colon, and only two in the rectum (*p*‐value < 0.001). Major differences were also observed regarding CMS status, where CMS2 was the dominating subtype in CLM and mLu, while a third of the PM cases were CMS4 (*p*‐value < 0.001). A large proportion (55% and 53% of the CLM and mLu cases, respectively) did not harbor hot‐spot mutations in either *KRAS* or *BRAF* (*p*‐value = 0.01), and only one of the CLM cases was *BRAF* mutated. OS from the time of metastasis surgery was median 85, 61, and 28 months for mLu, CLM, and PM patients, respectively (*p*‐value < 0.001).

### 3.2 Principal Component Analysis (PCA) and Assessment of Technical Variation

**FIGURE 1:**
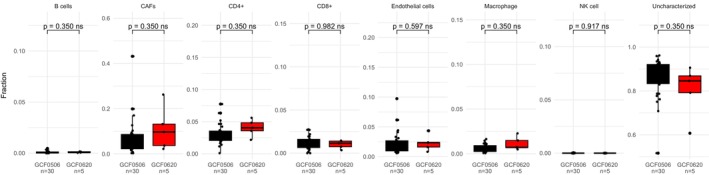
TME cell fractions for PM across datasets. Boxplots showing estimated TME cell fractions for PM samples between the two datasets, GCF0506 (*n* = 30) in black and GCF0620 (*n* = 5) in red. The “Uncharacterized” category represents the fraction of all characterized cells relative to the total number of non‐characterized cells present.

Ideally, one would attempt to pool data from different sequencing runs to increase the sample size and facilitate comparison of gene expression between different metastatic sites. As can be seen from Figure S1B, there were clear differences between the GCF0506 and GCF0620 sequencing runs. To analyze the potential impact of technical variation, the PM samples from both sequencing runs were explored. With PCA analysis we found that the five GCF0620 PM samples tended toward being outliers along the first two principal components relative to the 30 GCF0506 PM samples (Figure S1C). In addition, differential gene expression analysis comparing the two sequencing runs (Figure S1D) resulted in the identification of 396 differentially expressed genes. When comparing estimated TME cell fractions between PM samples from different sequencing runs based on gene expression data, although the differences did not reach statistical significance, discernible variations in the distributions of several cell types were evident (Figure 1). These findings collectively suggest that technical variation between sequencing runs may introduce spurious signals in the analysis. Therefore, subsequent comparative analyses of cell fractions were conducted within each batch to mitigate potential batch effects.

### 3.3 Associations Between TME Cell Composition and Metastatic Location

**FIGURE 2:**
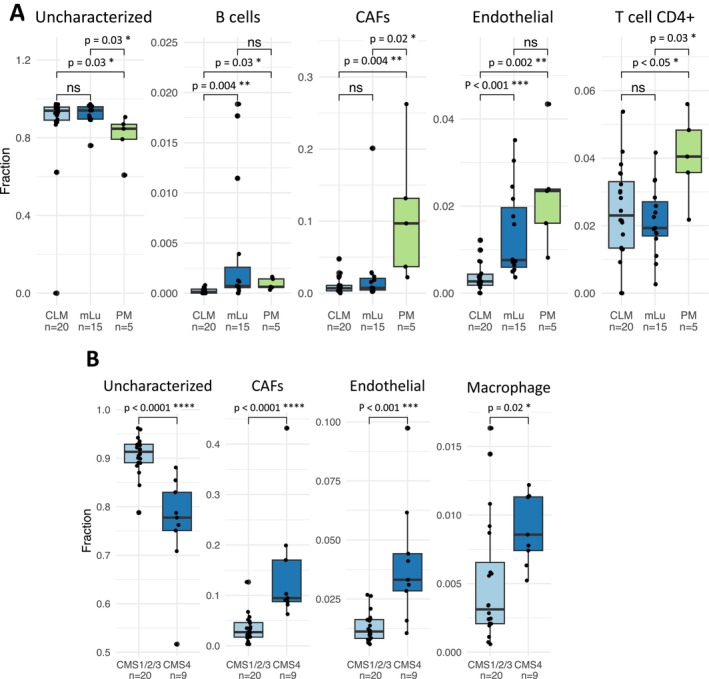
Cell composition in the TME of metastatic CRC. (A) Boxplot showing key findings from analysis of TME cell composition comparing different metastatic sites (GCF0620 dataset). *y*‐axis is the cell fractions, scaled according to individual cell type. (B) Boxplot showing key differences in the TME cell composition when comparing consensus molecular subtype (CMS) 1–3 to CMS4 in PM samples from the GCF0506 dataset. CAFs, cancer‐associated fibroblasts; CLM, colorectal liver metastasis; mLu, lung metastasis; PM, peritoneal metastasis; CMS, consensus molecular subtypes. ns, *p* > 0.05; *p ≤ 0.05; **p ≤ 0.01; ***p ≤ 0.001; ****, *p* ≤ 0.0001.

When comparing TME cell composition in samples from the different metastatic sites (GCF0620), the cell fractions were relatively similar, with the exception of four cell types for which a significant difference was retained after adjusting for multiple testing (Figure 2A). The B cell fraction was higher in mLu and PM compared to CLM (*p*‐value = 0.004 and 0.03, respectively), while the fraction of CAFs was higher in PM compared to CLM and mLu (*p*‐value < 0.004 and 0.02, respectively), and the endothelial cells fraction was higher in mLu and PM relative to CLM (*p*‐values < 0.001 and 0.002, respectively). An increased fraction of CD4+ T cells was also detected in PM compared to CLM and mLu (*p*‐value < 0.05 and 0.03, respectively).

### 3.4 Associations Between TME Cell Composition and CMS Subtypes

The majority of the CLM samples and all the mLu samples were classified as CMS2, while the PM samples were more diversely distributed between CMS1, CMS2, and CMS4 (Table 1). The CMS4 group has emerged as a particularly interesting subgroup in PM [23], and when comparing the cell composition of PM samples classified as CMS1‐3 with CMS4 (from GCF0506), the CMS4 subtype was shown to be enriched for CAFs, endothelial cells, and macrophages (*p*‐value < 0.0001, < 0.001 and 0.02, respectively, Figure 2B).

### 3.5 Increased EMT, TNF‐⍺ Signaling, and Angiogenesis in PM, Particularly in the CMS4 Subtype

**FIGURE 3:**
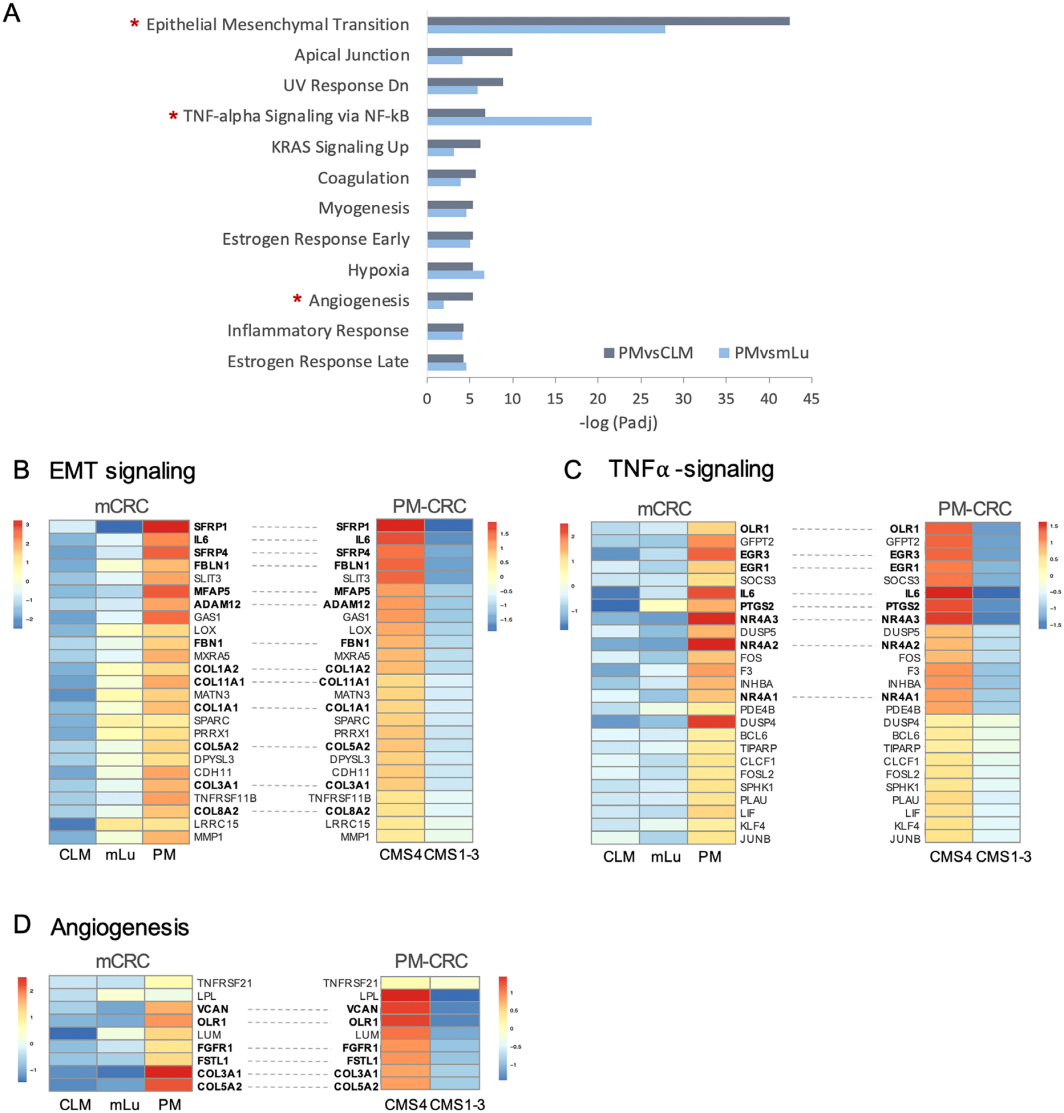
GSEA of differentially expressed genes. (A) GSEA showing signaling pathways that are affected by genes up‐regulated in PM compared to CLM (dark blue) and mLu (light blue). DEGs involved in EMT (B), TNF‐⍺ signaling (C), and angiogenesis (D) were enriched in PM, specifically the CMS4 subgroup. Heatmaps showing relative average gene expression levels of the top DEGs.

**FIGURE 4:**
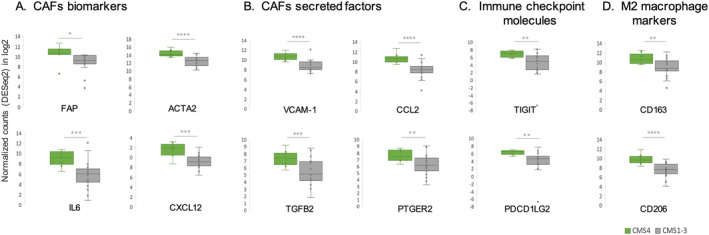
Differentially expressed genes related to CAFs, immune checkpoints, and macrophages in CMS4 vs CMS1‐3 PM‐CRC (GCF0506). Enrichment of additional CAF‐specific markers (A) and secreted factors (B), immune checkpoint molecules (C), and M2 macrophage markers (D) in PM CMS4 compared to CMS1‐3. Boxplots of selected genes indicating median, 25 and 75 percentiles. *p < 0.05, **p < 0.01, ***p < 0.001, ****p < 0.0001.

GSEA analysis of DEGs between the metastatic sites (GCF0620) revealed up‐regulation of genes involved in EMT (80 genes, Figure 3A,B), TNF‐⍺ signaling via NF‐kB (45 genes, Figure 3A,C), and angiogenesis (10 genes, Figure 3A,D) in PM compared to both CLM and mLu. The same genes were found to be enriched in the CMS4 subtype of PM (GCF0506) compared to CMS1‐3. Among the top up‐regulated genes involved in EMT were *IL6*, *SFRP1/4*, FBLN1, FBN1, MFAP5, ADAM12, and several collagens (Figure 3B). The transcription factors NR4A2/3 and EGR1/3, PTGS2 (COX2), OLR1 in addition to IL6, were among the top up‐regulated genes involved in TNF‐⍺ signaling (Figure 3C). Angiogenic factors that were enriched in CMS4 PM were *VCAN*, *FGFR1*, *FSTL1*, *OLR1*, and *collagen* (Figure 3D). Well‐known CAF markers, such as *FAP* and *ACTA2* (⍺‐SMA), as well as additional CAF secreted factors (*CXCL12, CCL2, TGFβ2, VCAM1*, PTGER2/ PEG2) were also found enriched in the PM CMS4 subgroup (Figure 4A,B). Finally, we also found increased expression of the immune checkpoint molecules *TIGIT* and *PD‐L2* (PDCD1LG2; Figure 4C) and M2 macrophage markers (CD163 and CD206) in this patient subgroup (Figure 4D).

## 4 Discussion

Only four cell types exhibited significant differences when comparing the three metastatic sites, CAFs, B cells, endothelial cells, and CD4^+^ T cells, giving the impression of a quantitatively similar metastatic tumor microenvironment, independent of the metastatic site. This is somewhat surprising, given that the liver, lungs, and peritoneum are very different in tissue composition and biological function and face different challenges in generating effective immune responses against pathogens [24]. However, although the estimated immune cell fractions were similar, there might be important differences in phenotypic properties, differentiation status, longevity, turnover rate, and regulatory mechanisms that cannot be addressed by the deconvolution tools. Thus, to fully understand the immune contexture of the different metastatic locations, additional studies are needed.

CMS classification showed that CLM and mLu were dominated by the canonical CMS2 subtype, while only one third of the PM tumors were CMS2. Resectable CLM were previously shown to be enriched for CMS2 by us and others [20, 25] while mLu have, to our knowledge, not been specifically characterized with respect to CMS. The PM cohort exhibited more diverse findings with a substantial proportion of the tumors classified as CMS1, which originally in pCRC were typically enriched for MSI, CpG island methylator phenotype, high frequency of *BRAF* mutations, and immune cell infiltration [19]. The PM tumors in our cohort were frequently *BRAF* mutated, but only 4 out of 35 tumors were MSI, and immune cell enrichment was observed in CMS4 rather than in the CMS1‐3 categories. Interestingly, a third of the PM tumors were classified as CMS4, which in pCRC was associated with mesenchymal features, TGF‐β activation, and angiogenesis [19]. High frequency of the CMS4 subtype in PM is in line with previous findings and may contribute to the poor prognosis and inferior treatment response associated with PM compared to CLM and mLu cohorts [23, 26].

The most distinctive finding separating the metastatic locations was the observed enrichment of CAFs in PM compared to CLM and mLu, and CAFs were also enriched in the CMS4 subset of the PM tumors. Mesothelial‐derived CAFs have been shown to be crucial for the establishment and metastatic progression in the peritoneum [27]. This represents a metastatic pathway that is distinct from the tumor cell seeding through the lymphatic or systemic circulation, which is the main mechanism for development of liver and lung metastases. Cancer cells reprogram normal fibroblasts into CAFs, initiating tumor‐favorable signaling, including pro‐EMT signaling through secretion of TGF‐β [28], as well as remodeling of extracellular matrix to make it more favorable for tumor escape and invasion and creating a barrier to immune cell infiltration and penetration of anticancer drugs [29]. This is in line with our findings of increased EMT signaling in PM, specifically in the CMS4 subgroup, where several proteins involved in this process are known to be secreted by CAFs (Figure 3) [30, 31, 32, 33, 34, 35]. CAF markers, such as FAP and ACTA2 (⍺‐SMA) [33], were also elevated in this subgroup, confirming the TME deconvolution findings (Figure 4).

CAFs may also promote tumor angiogenesis through several molecular mechanisms [36], which would be supported by the observed increase in endothelial cell fraction, notably in PM compared to CLM, and particularly within the CMS4 subgroup. Additionally, elevated levels of CAF‐secreted pro‐angiogenic factors, specifically in the CMS4 subgroup (Figure 2), further corroborate this association. Furthermore, CAFs may alter the anti‐tumor immune response through secretion of several immune modulators such as IL6, CCL2, CXCL12, and TGFβ2 [37], which we found to be up‐regulated in CMS4 PM. These factors may recruit and modulate CD4+ T cells, which also had higher levels in PM tissue, into Th2, Th17, or Tregs with tumor‐promoting responses, and may also increase the expression of immune checkpoint molecules on T cells/tumor cells, causing T cell exhaustion. In fact, the immune checkpoint molecules TIGIT and PD‐L2 were found up‐regulated in the same subset of patients, supporting this scenario. The same CAF‐secreted factors (CCL2, CXCL12, and IL‐6) could also promote recruitment and differentiation of monocytes into M2 macrophages [38] which corresponds well with increased fractions of macrophages in the PM CMS4 subgroup. Macrophages exhibit a broad range of phenotypes, where M1 (anti‐tumor) and M2 (pro‐tumor) represent the polar ends of the spectrum [7]. Although EPIC does not distinguish between macrophage phenotypes, we do see an up‐regulation of M2 markers in PM CMS4 subgroup, which might indicate increased presence of the pro‐tumorigenic phenotype that may contribute to angiogenesis and immune suppression.

**FIGURE 5:**
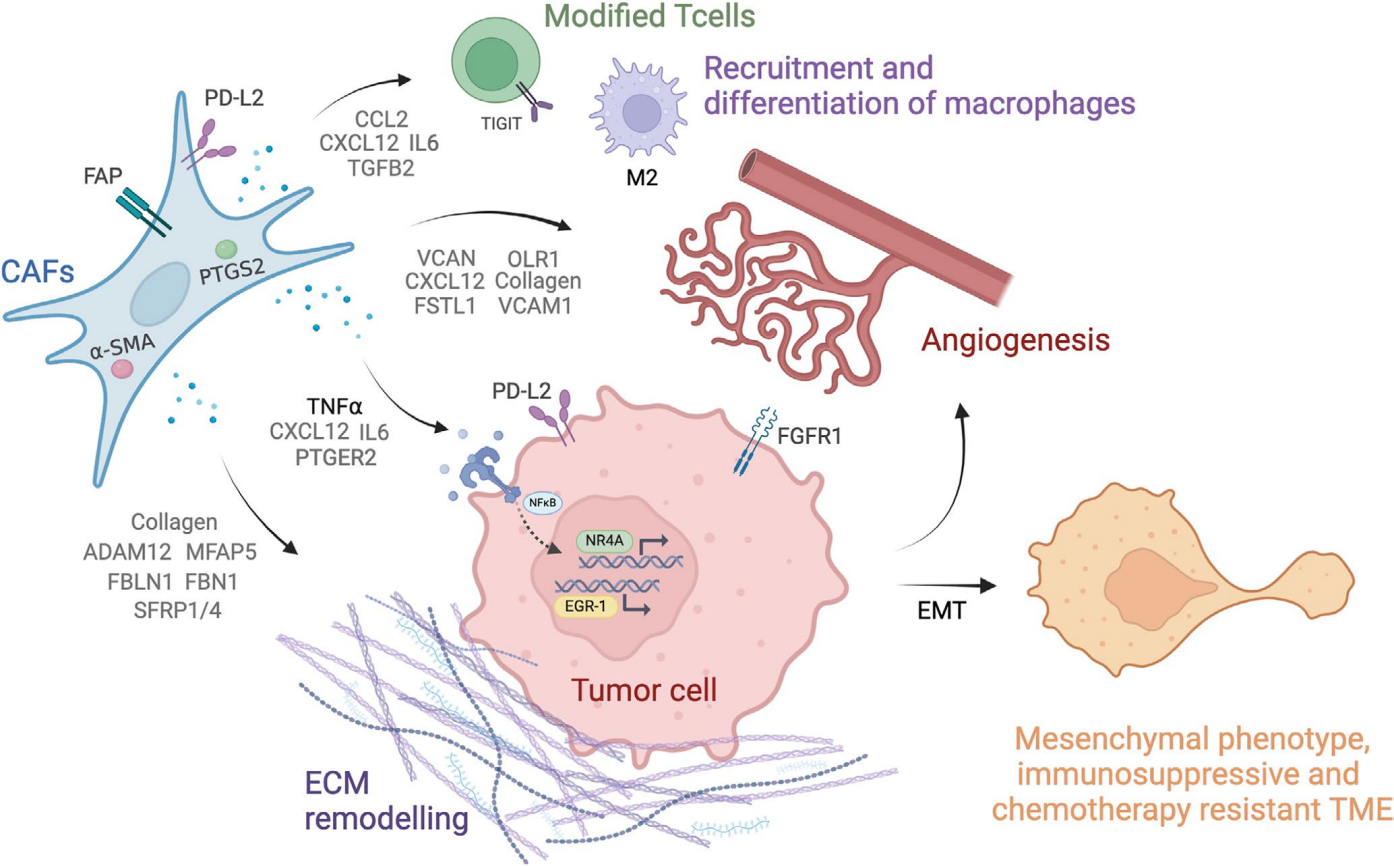
Graphical overview. CAFs and TNF‐⍺ signaling play a prominent role in the CMS4 subgroup of PM‐CRC, modulating the tumor cells into a mesenchymal‐like phenotype which could contribute to facilitate intraperitoneal spread, as well as creating an immunosuppressive and chemotherapy‐resistant TME. Created by BioRender.com.

In addition to EMT signaling and angiogenesis, TNF‐⍺ signaling via NF‐kB was enriched in the CMS4 subgroup of PM. This signaling pathway includes the up‐regulated transcription factors NR4A3 and EGR1/3, which are known regulators of cancer progression, EMT, angiogenesis, and inflammation [39, 40, 41]. The pathway may be activated by cytokines and growth factors secreted by CAFs, such as COX2 (PTGS2)/PEG2 (PTGER2) [42] or IL6 which were up‐regulated in PM CMS4, or through pathway‐related genetic mutations in the cancer cells, such as *KRAS* mutations [40]. NR4A is also known to promote CAF activity [39]. Thus, these results suggest that CAFs and TNF‐⍺ signaling play a prominent role in the CMS4 subgroup of PM‐CRC, modulating the tumor cells into a mesenchymal‐like phenotype to facilitate intraperitoneal spread, as well as creating an immunosuppressive and chemotherapy‐resistant TME (Figure 5). Interestingly, associations between CAFs and TNF‐⍺ signaling in CMS4 primary CRC have also been observed in a recent study by Leonard et al. [43].

As CAFs play an essential role in forming the PM‐CRC microenvironment, targeting CAFs and CAF‐signaling could be a therapeutic option, particularly in the CMS4 PM subtype. Potential interventions for CAFs include targeting surface markers such as FAP [44], DNA vaccines inducing CD8+ killing of CAFs, and CAR T cells engineered to target FAP [45, 46]. Targeting CAF signaling molecules, such as CXCL12 and IL‐6 [47, 48, 49], CAF‐associated pathways that regulate angiogenesis [36], and combination therapy of VEGF receptor inhibitors with mutated *BRAF* inhibitors are also promising strategies [50]. Another strategy is reprogramming immunosuppressive M2 macrophages into the immunoactive M1 phenotype through nanoparticle delivery of toll‐like receptor agonists [51]. Targeting TNF‐⍺/NF‐kB signaling and inhibiting NR4A or EGR1 receptors is another option [39, 52]. Therefore, targeting CAFs, M2 macrophages, and TNF‐⍺ signaling alone or with other anti‐tumor drugs could represent therapeutic strategies in PM, especially for the CMS4 subtype, as indicated by this work.

The main limitation of this study relates to the limited number of PM samples available in the comparison with CLM and mLu, due to observed batch effects when attempting to merge the two RNA seq datasets. Batch correction tools [53, 54, 55] are typically used to address such imbalances, but their application may introduce additional systematic biases into the data and should be performed cautiously [56]. Randomization of potential confounding factors for equal distribution across groups is a recommended approach [53, 57], but this was not feasible in this study, as the two sequencing runs were planned for separate projects. While cell deconvolution methods for bulk tissue can be reliable under ideal circumstances [10], the technical challenges associated with RNA‐seq remain limiting. While more recent cell deconvolution tools using single‐cell data as a reference are available [58], leveraging their full potential would necessitate having single‐cell reference data specific to the immune contexture in CLM, mLu, and PM. Alternative methods allowing spatial analysis at the transcriptomic or protein level may therefore be more appropriate for further investigations. Ongoing studies in our group using imaging mass cytometry aim to build upon the findings from this study, which provide a promising starting point for further research. Finally, this study does not investigate CAF subtypes as their classification is quite complex and controversial, and more advanced techniques such as single‐cell transcriptomics and spatial proteomics would be more suited to address this issue.

In conclusion, this study revealed insights into the tumor microenvironment of mCRC comparing different metastatic sites. TME deconvolution analysis of mRNA‐seq data generated from CRC liver, lung, and peritoneal metastases revealed broadly similar immune cell composition, but with some apparent differences, specifically enrichment of CAFs in the CMS4 PM microenvironment. CAF‐related secreting factors involved in EMT, angiogenesis, and immune modulation along with TNF‐⍺ signaling through NF‐kB were specifically enriched in PM exhibiting the CMS4 subtype, collectively promoting intraperitoneal spread, immune suppression, and chemotherapy resistance. Targeting CAF‐associated pathways, M2 macrophages, and TNF‐⍺ signaling could represent potential novel therapeutic approaches in PM‐CRC, a metastatic site that carries a particularly poor prognosis.

## Author Contributions

**Eirik Høye:** conceptualization (supporting), data curation (equal), formal analysis (equal), investigation (equal), software (equal), visualization (equal), writing – original draft (equal), writing – review and editing (equal). **Chakravarthi Kanduri:** data curation (equal), formal analysis (supporting), software (equal), writing – review and editing (supporting). **Annette Torgunrud:** resources (lead), writing – review and editing (supporting). **Susanne Lorenz:** methodology (lead), writing – review and editing (supporting). **Bjørn Edwin:** writing – review and editing (supporting). **Stein G. Larsen:** writing – review and editing (supporting). **Åsmund A. Fretland:** writing – review and editing (supporting). **Vegar J. Dagenborg:** resources (supporting), writing – review and editing (supporting). **Kjersti Flatmark:** conceptualization (lead), funding acquisition (lead), project administration (equal), resources (supporting), supervision (equal), writing – original draft (equal), writing – review and editing (equal). **Christin Lund‐Andersen:** conceptualization (supporting), formal analysis (equal), investigation (equal), project administration (equal), resources (supporting), supervision (equal), visualization (equal), writing – original draft (equal), writing – review and editing (equal).

## Ethics Statement

Norway's Regional Committees for Medical and Health Research Ethics (REK) approved the CLM (ID# 2011/1285/REK sør‐øst B), mLu (Ref: S‐05307), and PM (ID# 2010/2390/REK sør‐øst B) samples collection, respectively.

## Consent

Written informed consent was obtained from all participating patients.

## Conflicts of Interest

The authors declare no conflicts of interest.

## Supporting information


File S1.

File S2.

File S3.

File S4.

File S5.

File S6.

Figure S1.

## Data Availability

Tumor microenvironment deconvolution data and code are available at: https://github.com/eirikhoye/met_deconv. RNA‐seq and clinical data are stored in the European Genome‐Phenome Archive (EGA) data repository (accession number: EGA50000000615).
