# Supplementary material for: Enrichment of Cancer‐Associated Fibroblasts, Macrophages, and Up‐Regulated TNF‐α Signaling in the Tumor Microenvironment of CMS4 Colorectal Peritoneal Metastasis
Source: Cancer Med. 2024 Dec 30;14(1):e70521. doi: 10.1002/cam4.70521 (PMC11683539; doi:10.1002/cam4.70521)
Supplement: Supplementary file 7 — Figure S1. [file CAM4-14-e70521-s002.docx]

******

***Supplementary Figure 1.***

***A*** *Concordance analysis comparing the cell type prediction by EPIC, quanTIseq and CIBERSORT on the complete dataset. Bars show spearman r correlation coefficient of rank transformed cell fractions between the different deconvolution methods.*

***B*** *PCA plots generated from analysis of global mRNA expression. Individual samples are shown as small circles.*

***C*** *Principal component analysis of PM samples annotated by sequencing run. Individual samples are shown as small circles, while the large circles represent the mean of each feature. Red points represent GCF0620, blue points represent GCF0506.*

***D*** *Volcano plot of DESeq2 estimated fold changes comparing the two datasets. X axis is log2 transformed fold changes, positive means higher in GCF0620, y axis is -log10 transformed FDR adjusted p-value. Red points represent genes with adjusted p-value < 0.1.*
